# Morphological and physiological variation of soybean seedlings in response to shade

**DOI:** 10.3389/fpls.2022.1015414

**Published:** 2022-10-06

**Authors:** Yushan Wu, Ping Chen, Wanzhuo Gong, Hina Gul, Junqi Zhu, Feng Yang, Xiaochun Wang, Taiwen Yong, Jiang Liu, Tian Pu, Yanhong Yan, Wenyu Yang

**Affiliations:** ^1^College of Agronomy, Sichuan Agricultural University, Chengdu, China; ^2^Key Laboratory of Crop Eco-Physiology and Farming System, Sichuan Engineering Research Center for Crop Strip Intercropping System, Chengdu, China; ^3^Crop Research Institute, Chengdu Academy of Agricultural and Forestry Sciences, Chengdu, China; ^4^National Center of Industrial Biotechnology (NCIB), PMAS Arid Agriculture University, Rawalpindi, Pakistan; ^5^Plant and Food Research, Blenheim, New Zealand; ^6^College of Life Science, Sichuan Agricultural University, Chengdu, China; ^7^College of Grassland Science and Technology, Sichuan Agricultural University, Chengdu, China

**Keywords:** shade, soybean, plasticity, morphological, physiological

## Abstract

Soybean (*Glycine max*) is a legume species that is widely used in intercropping. Quantitative analyses of plasticity and genetic differences in soybean would improve the selection and breeding of soybean in intercropping. Here, we used data of 20 varieties from one year artificial shading experiment and one year intercropping experiment to characterize the morphological and physiological traits of soybean seedlings grown under shade and full sun light conditions. Our results showed that shade significantly decreased biomass, leaf area, stem diameter, fraction of dry mass in petiole, leaf mass per unit area, chlorophyll a/b ratio, net photosynthetic rate per unit area at PAR of 500 μmol m^–2^ s^–1^ and 1,200 μmol m^–2^ s^–1^ of soybean seedling, but significantly increased plant height, fraction of dry mass in stem and chlorophyll content. Light × variety interaction was significant for all measured traits, light effect contributed more than variety effect. The biomass of soybean seedlings was positively correlated with leaf area and stem diameter under both shade and full sunlight conditions, but not correlated with plant height and net photosynthetic rate. The top five (62.75% variation explained) most important explanatory variables of plasticity of biomass were that the plasticity of leaf area, leaf area ratio, leaflet area, plant height and chlorophyll content, whose total weight were 1, 0.9, 0.3, 0.2, 0.19, respectively. The plasticity of biomass was positively correlated with plasticity of leaf area and leaflet area but significant negative correlated with plasticity of plant height. The principal component one account for 42.45% variation explain. A cluster analysis further indicated that soybean cultivars were classified into three groups and cultivars; *Jiandebaimaodou*, *Gongdou 2*, and *Guixia 3* with the maximum plasticity of biomass. These results suggest that for soybean seedlings grown under shade increasing the capacity for light interception by larger leaf area is more vital than light searching (plant height) and light conversion (photosynthetic rate).

## Introduction

Intercropping, defined as the cultivation of two or more crop species simultaneously in the same field (Willey, 1979; Francis, 1989), is widely practiced by smallholder farmers across the world, and is attracting attention in the context of ecological intensification of agriculture (Vandermeer, 2011; Brooker et al., 2015; Tanveer et al., 2017). Compared with sole cropping, intercropping often leads to higher yield because of the full use of time, space, and resources (Willey, 1990; Keating and Carberry, 1993; Szumigalski and Van Acker, 2006; Malézieux et al., 2009; Raza et al., 2021). Adaptive plant morphological and physiological responses to intercropping environments are likely to contribute to the yield advantage (Li et al., 2021; Wang et al., 2021). It has been shown that plasticity of the shoot traits of wheat contribute significantly to the enhanced light capture in wheat-maize intercropping systems (Zhu et al., 2015, 2016). This suggests that phenotypic plasticity, which is defined as the ability of a genotype to alter its expressed trait values in response to environmental conditions (Bradshaw, 1965; Valladares et al., 2007; Sultan, 2010), may improve crop performance in intercropping systems. However, detailed information about the genetic differences in phenotypic plasticity in the context of intercropping is still lacking. Such information can be used as a guide in variety selection and breeding to optimize the benefits of intercropping systems (Zhu et al., 2016).

The cereal-legume system is one of the most common types of intercropping systems (Yu et al., 2015), and soybean is one of the most widely used legumes in these systems, which include maize–soybean (Wu et al., 2021) and sorghum–soybean (Ghosh et al., 2009). Due to the shorter plant height and late sowing time than maize and sorghum, soybean plants are often grown under shade conditions in these intercropping systems (Wu et al., 2022). Plants adapt to shade through either shade tolerance (Yang et al., 2018) or shade avoidance mechanisms (Valladares et al., 2007; Valladares and Niinemets, 2008; Niinemets, 2010). Shade tolerance mechanisms, which can help plants survive under low light conditions, increase light harvesting or light use efficiency. These mechanisms include increasing chlorophyll (Chl) content, increasing specific leaf area, and reducing the Chl *a/b* ratio (Givnish, 1988; Valladares and Niinemets, 2008; Niinemets, 2010). Shade avoidance mechanisms, which can help plants escape from shade and likely increase light capture, include responses such as enhanced stem and petiole elongation, higher dry mass allocation to the stem than to the leaf and root, and develop a small leaf angles (Ballaré, 1999; Smith, 2000; Franklin and Whitelam, 2005; Vandenbussche et al., 2005; Casal, 2012; de Wit et al., 2012).

Phenotypic plasticity plays a remarkable role in the ecological distribution and evolutionary diversification of plants (Enbody et al., 2021). The degrees of plasticity differ among species or populations from contrasting habitats. It has been reported that shade-grown *Impatiens capensis* possesses longer stems and internodes than its counterparts grown under full sunshine and that *I. capensis* populations originating from open habitats are more sensitive to shade than those from shade habitats (Dudley and Schmitt, 1995; Anten et al., 2009). Other studies comparing the plasticity of morphological and physiological variables in different species found that shade-intolerant species from light habitats are more sensitive to light and exhibit a greater degree of plasticity than shade-tolerant species from shade habitats (Valladares et al., 2000a,b; Portsmuth and Niinemets, 2007). Comparisons of the plasticity of different functional traits revealed that shade-intolerant species have relatively higher plasticity for physiological traits, but lower plasticity for morphological traits than shade-tolerant species (Valladares et al., 2000b; Li et al., 2012; Naseer et al., 2022). It has been pointed out that light-favoring plants have enhanced physiological plasticity for variables related to photosynthesis, while shade tolerant species rely on enhanced plasticity in light-harvesting traits (Valladares et al., 2002; Poorter et al., 2019).

It has been shown that soybean plants display a suite of shade-avoidance responses when co-grown with maize; these responses result in a lower photosynthetic capacity, an elongated stem, reduced branching, a higher lodging rate and lower yield (Yan et al., 2010; Su et al., 2014; Yang et al., 2014; Wu et al., 2022). Previously, we showed that there are genetic differences in the shade responses of two soybean varieties (Gong et al., 2015). However, it remains unclear how these responses reflect the inherent strategies for coping with shade, and which traits should be displayed in the ideotype for breeding soybean varieties with better performance in intercropping. The aim of this study was: (1) to characterize the phenotypic variation in morphological and physiological traits of soybean grown under both shade and full-light conditions; (2) to quantify the relationship between the biomass of soybean and morphological and physiological traits under the different light conditions; and (3) to develop approaches to establish an ideotype contributing to higher biomass accumulation under shade based on plasticity in morphological and physiological traits.

## Materials and methods

### Experimental design and field management

**FIGURE 1:**
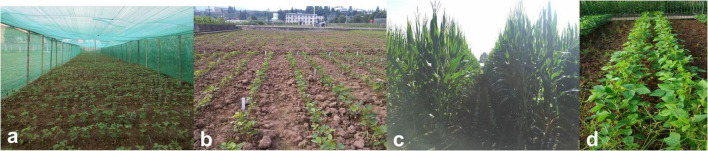
Photographes of the experiment. **(a)** Shade treatment in 2014; **(b)** full sun light treatment in 2014; **(c)** maize-soybean relay strip intercropping in 2015; **(d)** soybean monoculture in 2015.

**Experiment 1:** Experiment 1 was conducted in 2014 under field conditions at the Teaching and Experimental Farm of Sichuan Agricultural University in Ya’an (29°59’N, 103°00’E), which is located at the western border of the Sichuan Basin. The soil of the experimental field is a purple clay loam (pH 7.5). On June 19th, the seeds of 20 soybean varieties (names refer to Supplementary Table 1) were sown, each varieties were sown in one plot with a row spacing of 0.5 m and a row length of 2.5 m, and three rows of each variety were planted for each condition. The shade and full sun light treatments were started immediately after sowing. The shade treatment was achieved by installing green shading nets above the experimental field at a height of 2 m, ∼40% transmittance, PAR was around 500 μmol m^–2^ s^–1^, average daily temperature was 30.7 °C, average daily humidity was 71.9%. The average daily temperature and humidity of full sun light treatment was 32.9 °C and 64.5% (Yang et al., 2014; Wu et al., 2017b; Figures 1A,B). After the first trifoliolate leaves expanded, soybean seedlings were thinned to 0.1 m between plants in each row.

**Experiment 2:** In order to further verify the shade response of soybean and variety performance of 2014 in a real intercropping shade environment, we conducted Experiment 2 in 2015 at the same field of 2014, so the soil of the experimental field is a purple clay loam (pH 7.5). It was conducted by a split-plot experiment design with three replications. The planting pattern was set as main plot with two levels: maize-soybean relay strip intercropping (shade) and soybean monoculture (full sun light) (Figures 1C,D). 14 soybean varieties (in order to reduce the field work, we only selected 14 varieties with large difference in growth in shade) were used in sub plot based on the results of 2014 (Supplementary Table 1). The field arrangement of relay intercropping was carried out as follows. Briefly, For the full sun light treatment in sole cropping, soybeans were planted as solid rows with a 0.5-m row spacing. For the shade treatment in intercropping, soybean and maize were planted as alternating strips, and every soybean strip was relay intercropped between the maize strips. Each plot contained two maize and two soybean strips, and each soybean strip and maize strip consisted of two soybean and two maize rows. The strip spacing (distance between maize and soybean rows), soybean row spacing and maize row spacing were all 0.5 m (Wu et al., 2017b). Each plot was 6 m long. The maize cultivar, *Zhenghong 505*, was sown on 9 April and harvested on 9 August, the soybeans were sown on 20 June and harvested on 23 October. When soybean was planted, maize was 2.5 meters high and reduced the light interception rate of soybean by 66%, the average daily temperature and humidity of intercropping treatment was 30.1 °C and 68.6%. The weather condition (rainfall and temperature) of two years are shown in Supplementary Figure 1.

### Measurements

In Experiment 1, three individual plants grown in the middle section of the middle row were tagged on August 5th, 2014 (47 days after sowing); these plants were used for all measurements and each individual plant was a biological replicate. Photosynthetic indexes were mearsured on on the 47 days after sowing, and the middle leaflets of the latest fully expanded trifoliolate leaves were used for measurements. Photosynthesis was measured using a portable photosynthesis system (LI-6400XT, Li-Cor Inc., USA) equipped with an LED Light Source (6400-02B). Net photosynthetic rate per unit area was measured under light intensities of 500 (P_*N*500_) and 1,200 (P_*N*1200_) μmol m^–2^ s^–1^, a CO_2_ concentration of 380 μmol mol^–1^ sample, an air flow rate of 500 ml min^–1^, 60–75% relative humidity and a temperature of 30°C. The net photosynthetic rate value was taken when the range is between 0.1 and 0.2 after 1–2 min. After the measurement of photosynthesis, the same trifoliolate leaves were collected in white ziplock bag, and put in a ice box and brought to the laboratory no more than 30 min. First, leaves were scanned using a flatbed scanner (CanoScan LiDE 200, Canon Inc., Japan), and the captured images were used for later analysis of the leaflet area in ImageJ 1.45s. Second, two leaf discs (diameter = 1 cm) from the middle leaflet were punched out, and extracted in a centrifuge tube with 80% aqueous acetone solvent for 24 h in a dark environment with 20 °C indoor temperature, and then used spectrophotometer to determine the total Chl concentration and the Chl *a/b* ratio (Lichtenthaler, 1987). The remaining leaves were oven-dried and weighed to calculate the leaf mass per unit area and the Chl concentration per unit dry mass. Dried leaves were finally ground into a fine powder for the measurement of nitrogen (N) and carbon (C) concentration using an elemental analyzer (CE-440 Elemental Analyzer, Exeter Analytical Inc., USA). N concentrations were expressed based on unit content per unit area.

On August 6th, 2014 (48 days after sowing), the aboveground parts of the tagged plants were sampled and brought back to the laboratory to measure biomass and morphological traits. Plants were divided into three parts: stem, lamina and petiole. Laminas were scanned, and then the total leaf area per plant was determined using ImageJ. Plant height and stem diameter (measured at the middle point of the first internode) were also measured. The separate parts of the leaf were oven-dried at 70°C to a constant weight (∼72 h). Biomass, fraction of dry mass in the stem, fraction of dry mass in the lamina, and fraction of dry mass in the petiole were then calculated.

In Experiment 2, all sampling and measurement were conducted on July 27th, 2015 (37 days after sowing). First, P_*N*1200_ was measured on five newly expanded leaves in five individual plants grown in the middle section of the middle row per plot, other parameters were maintained as described in experiment 1. Second, the measured plant for photosynthetic rates were sampled and brought to laboratory, leaf area, plant height, stem diameter, biomass were measured as described in experiment 1. The mean value per plot were calculated and used for statistics.

### Data processing and analysis

The plasticity of plants in response to shade was calculated as the dimensionless slope of norm of reaction as previously reported (Sadras et al., 2009). Briefly, Plasticity = (*T_*i*_*shade*_*-*T*_*i_fulllight*_)/(*_*shade*_*-*_*fulllight*_*), where *T*_*i*_ is the value of one trait (e.g., biomass) of the *i*th variety, and is the mean value of the trait across all varieties. Thus, slope = 1 indicates average plasticity over the two light environments, slope > 1 indicates above-average plasticity, and slope < 1 indicates below-average plasticity (Sadras et al., 2009).

Two-way ANOVA was used to test for the effects of light and variety on the measured traits. Light was set as a fixed factor, and variety was set as a random factor. Before analysis, trait values were transformed by taking the natural logarithm. Correlation and regression analysis were used to explore associations between traits and plasticity (Supplementary Tables 2, 3 and Supplementary Figures 3, 4). Analyses were performed using *SPSS 19.0* software (*SPSS*, Chicago, USA).

**FIGURE 2:**
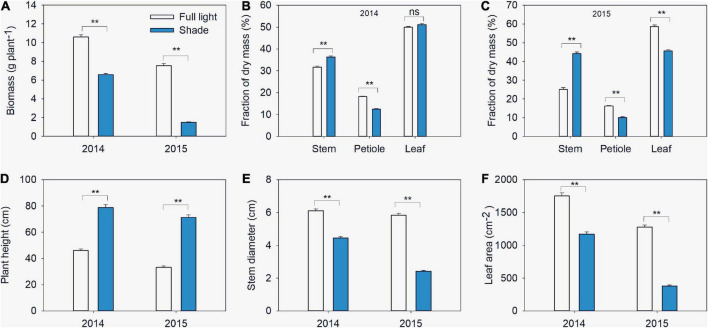
Biomass **(A)**, fractions of dry mass in stem, petiole and leaf **(B,C)**, plant height **(D)**, stem diameter **(E)** and leaf area **(F)** of different soybean varieties grown under shade and the control conditions in 2014–2015. Traits were expressed as mean ± standard errors from 20 cultivars in 2014 and 14 cultivars in 2015. ** represent significant difference at 0.01 level.

**FIGURE 3:**
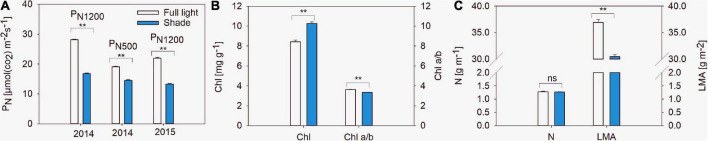
Net photosynthetic rates at 500 μmol m^– 2^ s^– 1^ (P_N500_) and 1,200 μmol m^– 2^ s^– 1^ (P_N1200_) **(A)**, chlorophyll concentration (Chl) and Chl a/b ratio (Chl a/b) **(B)**, nitrogen (N) concentration and leaf mass per unit area (LMA) **(C)** of 20 soybean varieties grown under shade and the control conditions in 2014. Traits were expressed as mean ± standard errors from 20 cultivars in 2014 and 14 cultivars in 2015. ** represent significant difference at 0.01 level.

R version 4.0.5 was used to reveal the quantitative relationships between the plasticity of biomass and the plasticity of morphological and physiological traits. Features selection was performed with the *train* function in R package *caret* v6.0-91. Then, features importance was obtained with *varImp* function in R package *caret* v6.0-91 (Figure 2). The critical features (*p* < 0.05) were selected, and correlation analysis was performed with *ggpairs* function in R package *GGally* v2.1.2 (Figure 3). Similarly, the critical features were performed with principle component analysis with the *PCA* function in R package *FactoMineR* package v1.34. The results of PCA were visualized with the *fviz_pca_biplot* function in R *factoextra* package v1.0.7 (Figure 6A). Meanwhile, hierarchical cluster analysis (Figure 6B) was performed with the *hclust* function and visualized with the *fviz_dend* function in R package *factoextra*.

## Results

### Plant biomass and biomass allocation

In 2014, shade significantly reduced biomass, the mean biomass of soybean seedlings grown under shade across all varieties was 6.6 g per plant, which was 37.8% less than that of seedlings grown under full light (10.6 g per plant) (Figure 2A). Similar variations trend were observed in 2015 when soybean plants grown under shade in intercropping and under full light in sole cropping. The mean biomass of soybean seedling was 1.5 g per plant, the corresponding grown under full light was 7.6 g per plant (Figure 2A). Significant interactions between light and variety were found for biomass. Partitioning of total sum of squares (SS) indicated that the light effect was the major contributor (Table 1).

**TABLE 1:** The sum of squares (SS) and mean squares (MS) of two-way ANOVAs of the effects of light and variety on morphological traits.

Year	ANOVA	d.f.	BMS		f_s_		f_p_		f_l_		LA		PHT		DMT	
			SS	MS	SS	MS	SS	MS	SS	MS	SS	MS	SS	MS	SS	MS
2014	Light	1	6.57	6.57**	0.56	0.56**	4.45	4.45**	0.01	0.01	4.93	4.93**	9.01	9.01**	3.00	3.00**
	Variety	19	2.75	0.14**	0.91	0.05**	0.90	0.05**	0.36	0.02**	3.39	0.18*	4.47	0.24**	0.05*	0.05*
	Light*Variety	19	0.92	0.05**	0.11	0.01**	0.15	0.01*	0.06	0.00**	1.23	0.06**	0.7	0.04**	0.02**	0.02**
2015	Light	1	56.93	56.93**	∼	∼	∼	∼	∼	∼	32.42	32.42**	12.31	12.31**	16.42	16.42**
	Variety	13	1.84	0.14	∼	∼	∼	∼	∼	∼	1.69	0.13	1.947	0.15*	0.61	0.04
	Light*Variety	13	1.68	0.12**	∼	∼	∼	∼	∼	∼	1.47	0.11**	0.56	0.04**	0.39	0.03**

*, ** represent significant difference at 0.05 and 0.01 levels, respectively. BMS, biomass; f_s_, fraction of dry mass in stem; f_p_, fraction of dry mass in petiole; f_l_, fraction of dry mass in lamina; LA, leaf area per plant; PHT, plant height; DMT, diameter of the first node.

In 2014, the mean fraction of dry mass in stem increased from 31.7% under full light to 36.4% under shade. Meanwhile, the mean fraction of dry mass in petiole decreased from 18.3% under full light to 12.5% under shade (Figure 2B). There was no difference in fraction of dry mass in leaf between plants grown under the two treatments. Significant varietal differences and interactions between light and variety were found in fraction of dry mass. The total SS indicated that the variety effect was the major contributor for stem and leaf, while petiole variation was caused by light (Table 1). In 2015, Similar variations trend of fraction of dry mass in stem and petiole were observed. While the mean fraction of dry mass in leaf decreased from 58% under full light to 45% under shade.

### Morphological traits

In 2014, plant height greatly increased under shade conditions, while stem diameter decreased. The mean plant height of soybean seedlings grown under shade across all varieties was 79 cm, which was 71.5% higher than that for seedlings grown under full light (46 cm), while the mean stem diameter reduced from 6.1 mm in full light to 4.5 mm in shade (Figures 2D,E). leaf area was significantly reduced in shade. The mean value of leaf area across all varieties grown under shade was 1,171.1 cm^2^, which was 33.3% less than that under full light (1,755.4 cm^2^) (Figure 2F). In 2015, when grown under shade in intercropping, morphological traits significantly varied. Mean plant height of soybean seedling was 71.3 cm, but the value of seedlings grown under full light was 33.3 cm (Figure 2D). Meanwhile, the leaf area of soybean seedlings reduced from 1,278.4 cm^2^ in full light to 379.9 cm^2^ in shade, and the stem diameter reduced from 5.84 mm in full light to 2.42 mm in shade (Figures 2E,F). Significant interaction between light and variety were found for those traits in both two years, the partition of the total SS revealed that the major percentage was attributable to light (Table 1). The yields of the 14 soybean varieties in two treatments are also shown in Supplementary Table 4.

### Physiological traits

Leaf physiological traits related to light use efficiency were affected by shade. Leaf mass per unit area, P_*N*500_, P_*N*1200_ and the Chl *a/b* ratio decreased under the shade condition, while Chl content increased (Figure 3). Leaf mass per unit area and Chl content differed between varieties, while P_*N*500_, P_*N*1200_ and leaf nitrogen content did not. The interactions between light and variety for leaf mass per unit area, P_*N*500_, P_*N*1200_, Chl content, Chl a/b ratio and N content were significant. ANOVA results revealed that light effect was the major contributor for Leaf mass per unit area, P_*N*500_, P_*N*1200_, Chl a/b ratio and nitrogen content, while Chl content variation was caused by variety (Table 2).

**TABLE 2:** The sum of squares (SS) and mean squares (MS) of two-way ANOVAs of the effects of light and variety on physiological traits.

Year	ANOVA	d.f.	LMA		Pn500		Pn1200		Chl		Chl a/b		N	
			SS	MS	SS	MS	SS	MS	SS	MS	SS	MS	SS	MS
2014	Light	1	1.10	1.10**	2.29	2.29**	8.09	8.09**	1.23	1.23**	0.23	0.23**	0.00	0.00
	Variety	19	0.73	0.04*	0.54	0.03	0.33	0.02	1.59	0.08*	0.14	0.01**	0.39	0.02
	Light*Variety	19	0.32	0.02**	0.28	0.01	0.26	0.01**	0.58	0.03**	0.03	0.00*	0.33	0.02**

*, ** represent significant difference at 0.05 and 0.01 levels, respectively. LMA, leaf mass per unit area; Pn_500_, net photosynthetic rate per unit area at 500 μmol m^–2^ s^–1^; Pn_1200_, net photosynthetic rate per unit area at 1,200 μmol m^–2^ s^–1^; Chl, chlorophyll content per unit dry mass; Chl a/b, chlorophyll a/b ratio; N, nitrogen content per unit area.

### Relationships between the biomass and the morphological and physiological traits

In 2014, the biomass of soybean seedlings was positively correlated with leaf area (Supplementary Figure 3A), as well as with the stem diameter under both shade and full light conditions (Supplementary Figure 3B). But biomass was not correlated with plant height (Supplementary Figure 3C), P_*N*500_ (Supplementary Figure 3E) and P_*N*1200_ (Supplementary Figure 3F). Plant height was positively correlated with f_*S*_ under both shade and full light conditions (Supplementary Table 2). Interestingly, leaf mass per unit area was negatively correlated with biomass only under the shade condition (Supplementary Figure 3D).

In 2015, for the variation between shade in intercropping and full light in sole cropping, biomass of soybean seedlings positively correlated with leaf area (Supplementary Figure 4A). For the relationship between biomass and plant height, negative correlations were found only in shade conditions (Supplementary Figure 4B).

### Quantitative relationships between the plasticity of biomass and the plasticity of morphological and physiological traits

**FIGURE 4:**
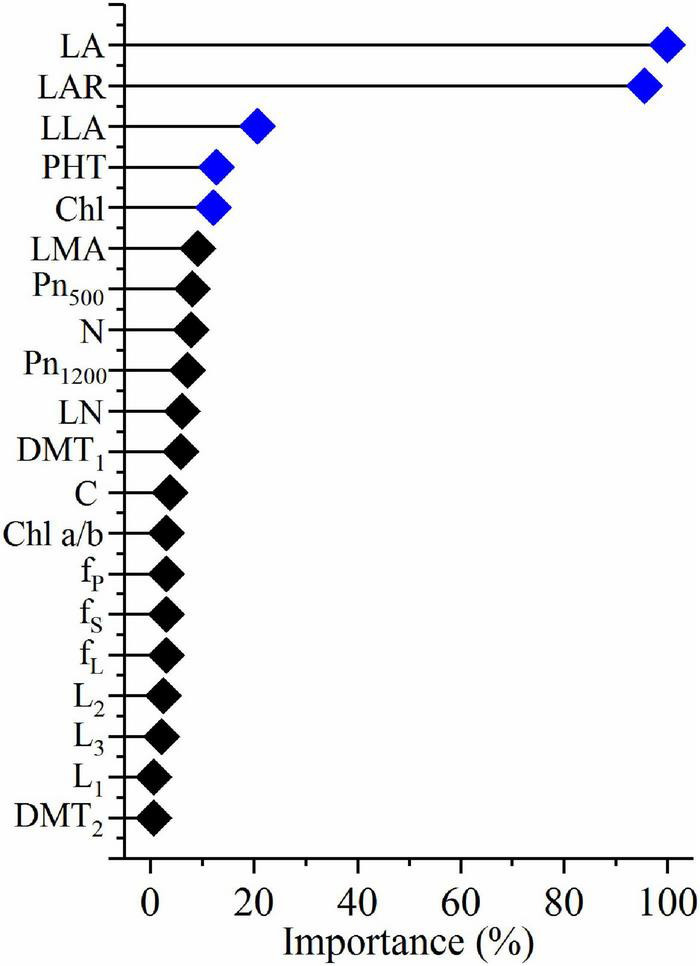
Relative importance of the plasticity of morphological and physiological variables that affect the plasticity of biomass in shade. LA, leaf area per plant; LAR, leaf area ratio; LLA, leaflet area; LN, leaflet number; PHT, plant height; Chl, chlorophyll content per unit dry mass; Chl a/b, chlorophyll a/b ratio; LMA, leaf mass per unit area; Pn_500_, net photosynthetic rate per unit area at 500 μmol m^– 2^ s^– 1^; Pn_1200_, net photosynthetic rate per unit area at 1,200 μmol m^– 2^ s^– 1^; N, nitrogen content per unit area; LN, leaflet number; C, carbon content per unit area; DMT_1_, diameter of the first node; DMT_2_, diameter of the second node; f_S_, fraction of dry mass in stem; f_P_, fraction of dry mass in petiole; f_Lr_, fraction of dry mass in lamina; L_1_, first internode length; L_2_, second internode length; L_3_, third internode length.

**FIGURE 5:**
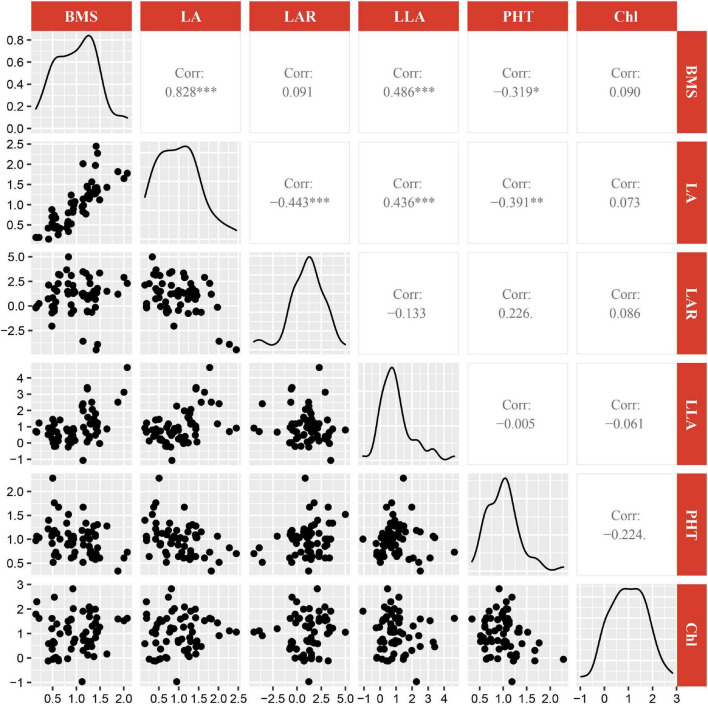
Correlations between the plasticity of biomass and the plasticity of top five most important explanatory variables. BMS, biomass;LA, leaf area; LAR, leaf area ratio; LLA, leaflet area; PHT, plant height; Chl, Chl content. *, **, *** represent significant difference at 0.05, 0.01, and 0.001 levels, respectively.

**FIGURE 6:**
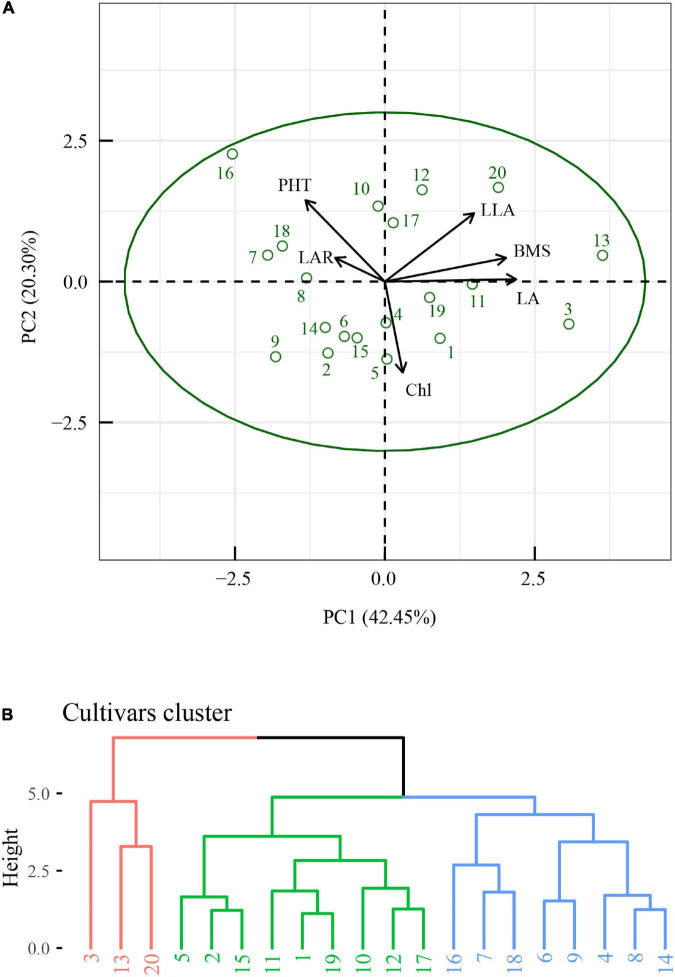
Principal component analysis (PCA) of the plasticity of biomass and the plasticity of top five most important explanatory variables **(A)**, and clustering analysis of the soybean cultivars **(B)**. BMS, biomass; LA, leaf area; LAR, leaf area ratio; LLA, leaflet area; PHT, plant height; Chl, Chl content. The numbers in panel **(B)** represent cultivars.

A multi-variable analysis and features selection were conducted to evaluate the relative importance of plasticity variables of biomass. Results indicated that the plasticity of leaf area, leaf area ratio, leaflet area, plant height, and Chl content were the top five explanatory variables, which significantly affected the plasticity of biomass (Figure 4). A further pearson correlations between the plasticity of biomass and the plasticity of the top five explanatory variables indicated that only leaf area, leaflet area, and plant height were significantly positively correlated with biomass (Figure 5). However, leaf area and leaflet area were significant positive correlated with biomass, plant height was significant negative correlated with the biomass (Figure 5). Results of principal component analysis (PCA) showed that the relationships between soybean cultivars’ biomass and explanatory variables, i.e., leaf area, leaflet area, Chl content, leaf area ratio, and plant height. A total of 62.7% variation explained by the explanatory variables and the principal component one account for 42.5% variation explain (Figure 6A), this was constant with the results of features selection (Figure 4) and correlation analysis (Figure 5). On the basis of the top five explanatory variables, cluster analysis indicated that soybean cultivars were classified into three groups (Figure 6B). Cluster one, including cultivars 3 (*Jiandebaimaodou*), 13 (*Gongdou 2*), and 20 (*Guixia*), with the maximum plasticity of biomass.

## Discussion

### Soybean seedling responses to shade

Compared with seedlings grown in full light, shade-grown soybean seedlings showed increased plant height, internode length and fraction of dry mass in stem and reduced leaf area, leaflet area and leaflet number (Figure 2). These phenotypes are typical shade avoidance symptoms that allow plants to search for light and escape from shade (Franklin, 2008). The presence of these symptoms indicates that soybean seedlings escape from shade by increasing stem elongation at the cost of reduced leaf expansion (Valladares et al., 2011). We also found that plant height was positively correlated with fraction of dry mass in stem under both light treatments and that fraction of dry mass in stem was inversely related to fraction of dry mass in lamina (Supplementary Table 2). These results indicate a trade-off between increased plant height which allows plants to search for light, and leaf expansion which increases light capture and utilization. Under shading this trade-off was balanced toward light searching.

Other findings in this study were that biomass was positively correlated with stem diameter under both light conditions (Supplementary Figures 3B and Supplementary Table 2) and that the plasticity of biomass was correlated with the plasticity of stem diameter under shading net (Supplementary Table 3). In addition, the stem diameter was positively correlated with leaf area under both light conditions, but it was negatively correlated with the length of the first internode under shade. Because the measurement of stem diameter was taken at the middle point of the first internode, these results indicate that the growth in the horizontal direction led to an increase in internode length in seedling development, which was consistent with Zhang et al. (2020). As the trifoliolate leaves of soybean develop at the apex of stem, a wider stem might lead to bigger leaf primordia due to the presence of more cells produced by cell division (Reinhardt et al., 2005; Wu et al., 2017a). Whether the thicker stem could produce larger leaf and the stem diameter of seedling grown under shade could be used to predict the leaf area still need to be testified in future studies.

Besides avoiding shade via stem elongation, plants often cope with shade by increasing light use efficiency via a shade tolerance strategy (Gong et al., 2015). We found that a reduced Chl *a*/*b*, a lower leaf mass per unit area and a higher Chl concentration were beneficial for photosynthesis under low light conditions, consistent with previous studies (Hussain et al., 2020; Tan et al., 2022). A higher Chl concentration indicates that there are more pigment-binding proteins for photons captured by photosystem II per unit N content. As the light-harvesting complex of photosystem II contains mostly Chl *b*, increased accumulation of the light-harvesting complex under shade causes a decline in the Chl *a*/*b* ratio (Boardman, 1977; Anderson, 1986; Evans, 1989).

Leaf mass per unit area reflects the trade-off between the functions of the leaf lamina in light radiation interception and conversion. A decline in leaf mass per unit area is beneficial for receiving more light per unit of leaf mass; however, low leaf mass per unit area negatively affects carboxylation because the reduced thickness of the palisade mesophyll resulting in less area for CO_2_ exchange (Terashima et al., 2001, 2006, 2011). Leaf mass per unit area is also strongly correlated with leaf thickness (especially palisade thickness) and the chemical composition of the leaf (i.e., N content and Chl concentration) (Poorter et al., 2009). In this study, leaf mass per unit area was positively correlated with P_*N*1200_ (*r* = 0.572^**^) under full light, indicating that a thicker leaf mesophyll layer leads to increased CO_2_ exchange as discussed above. But, we did not find a relationship between leaf mass per unit area and P_*N*500_ when soybean was grown under the shade treatment, which suggesting that when leaf mass per unit area was reduced, photosynthetic rate of all soybean genotypes declined to the similar levels. It is notable that the biomass of shade-grown soybean was negatively correlated with leaf mass per unit area (Supplementary Figure 3D), suggesting that soybean seedlings respond to shade by increasing leaf mass per unit area, which increases light interception at the cost of light conversion.

Although the plasticity of plant morphological and leaf physiological traits were helpful for acclimating to shade, the biomass of soybean seedlings was still severely reduced under the shade treatment (Figure 2A). The plasticity of biomass was positively correlated with the plasticity in leaf area and stem diameter under both light treatments (Supplementary Table 3), although leaf area and stem diameter were significantly reduced under the shade condition (Figures 2E,F). However, the plasticity of biomass was not correlated with plant height, P_N500_, P_N1200_, or Chl concentration in the present study. Thus, the increase in plant height and Chl concentration, as well as the decrease in Chl *a/b* ratio could not offset the light deficit. Based on these findings, we speculate that the formation of a canopy that increases light interception is more important than increasing light conversion for soybean seedlings that cannot escape from shade.

Phenotypic plasticity in response to light is the remarkable ability of plants to adjust morphology and physiology under different light conditions (Delagrange et al., 2004; Valladares and Niinemets, 2008). Previous studies have found that mean plasticity in morphological traits in response to light (e.g., elongation of the stem and internodes) is lower for shade-tolerant plants than for shade-intolerant plants (Dudley and Schmitt, 1995; Valladares et al., 2000b; Sánchez-Gómez et al., 2006; Portsmuth and Niinemets, 2007). Most plants from open habitats show shade avoidance responses, such as searching for light and escaping shade, when grown under shade (Gommers et al., 2013). Therefore, plasticity in plant height and internode length could be explained as the capacity for light searching. In this study, all soybean varieties showed the capacity for light searching via stem elongation. However, the plasticity of biomass was negative correlated to the plasticity of plant height (Supplementary Table 3). This is probably because soybean seedlings could not escape from shade in our shading net treatment or intercropping. Thus, the elongation of the stem could not enhance light interception. It can be inferred that under intercropping conditions where there is a large companion crop, e.g., maize, a strategy that enhances leaf area and allows more light to be captured, would be more beneficial than escaping shade through stem elongation.

Leaves are involved in both shade avoidance and tolerance strategies, as the leaf lamina directly captures light and converts the light into carbohydrates. Leaf area as the most important factor that affect plasticity of biomass and has strong positive relationship between plasticity of biomass, suggesting that light capture by the leaf determines light utilization, rather than the light search by elongating the stem. The abundance of climbing plants in deep shade was found to be directly related to their ability to intercept light efficiently but not to their ability to increase carbon fixation by increasing leaf area (Valladares et al., 2011). Hence, these findings suggest that capacity for light capture and absorption by the leaf is different from the ability to search for light by elongating the stem under deep shade, although both strategies might help increase light capture.

### Phenotypic plasticity of light searching, capturing, and conversion and their relationships to agronomic practices

Soybean is a light-favoring crop and is usually grown in a sole cropping system without shade from other plants. Soybean seedlings display a suite of architectural and physiological changes in response to shade, and there are genetic differences in these responses as shown in this study. In the maize-soybean relay strip intercropping system, soybean grew under shade environment during the seedling stages and the morphological traits were more plastic. Our previous study pointed out that most of the genetic differences in morphological traits are only expressed during the seedling stages (Hussain et al., 2020). So, we infer that plant seedlings have higher plasticity in architectural and morphological traits than in leaf physiological traits. It has been reported that plasticity in architectural traits leads to increased light interception and a yield advantage in intercropping systems (Zhu et al., 2015, 2016; Li et al., 2021),but it depends on the planting configurations (Li et al., 2020). This previous finding, combined with our results, indicates that the performance of component crops in intercropping systems might be based on light capture rather than photosynthesis and that increasing light capture might be a more feasible approach for increasing the total light intercepted in field production.

Plasticity in leaf physiological traits, such as photosynthetic rates and Chl concentration, is associated with changes in the capacity for photosynthesis in the leaf mesophyll. However, the plasticity of leaf physiological traits were not correlated with plasticity of biomass. Among the 20 varieties that we investigated in experiment 1, there was less variation in photosynthetic rate, Chl content and Chl *a*/*b* ratio compared with morphological traits, consistent with the findings of our previous study on two soybean varieties (Gong et al., 2015; Wu et al., 2022). These findings suggest that the capacity to adjust photosynthetic rate seems to be a conserved resource use strategy (Valladares et al., 2000a); thus, the soybean genotypes with contrasting capacities for light conversion in the mesophyll did not differ in their ability to accumulate biomass. Therefore, the selection of soybean varieties possessing higher photosynthesis rates might have little effect on improving shade tolerance.

The evolution of crops is not only driven by natural forces but also by selection by humans to meet the demand for food; crops became less shade tolerant over the past few thousands of years when grown in sole cropping systems, and fewer studies have focused on the genotypic differences in the shade tolerance of crops. It had been reported that shade reduced tillering in maize (Rotili et al., 2021), leaf size and morphology in tomato (Chitwood et al., 2015) and also negatively affected biomass in grasses (Warnasooriya and Brutnell, 2014). Many shade avoidance on crops focused on discovery and validation of molecular function. Cultivated soybean was domesticated from its wild progenitor *Glycine soja*, which is a typical climbing species that usually grows in shade environments. It is obvious that cultivated soybean was domesticated by humans to grow in high-light conditions. However, the capacities for light searching, light capture and light conversion were maintained in soybean. When modern soybean is grown under shade conditions such as those found in intercropping systems, elongation of the main stem seems to be an atavism.

The responses of the investigated traits in this study can be classified into three functional strategies: light searching, light capture and light conversion. Among these strategies, increasing light searching via elongation of the stem easily led to lodging (Liu et al., 2016), and increasing light conversion by increasing Chl content and photosynthesis in the mesophyll could not offset the light limitation and seems to be a conserved response among genotypes (Gong et al., 2015). The strategy of searching for light via stem elongation might increase the opportunity to escape from shade or to intercept more light; however, this trait is probably not acceptable in agriculture for several reasons. Firstly, it is hard for soybean plants to escape from shading in intercropping systems due to the presence of a tall companion crop, e.g., maize (Fan et al., 2018). Thus, enhanced stem elongation will not contribute to biomass accumulation. Secondly, over-elongated plants have increased rates of lodging (Liu et al., 2015, 2016). Soybean varieties with less stem elongation have a lower capacity to search for light. As we found negative correlation between the plasticity of biomass and plasticity of plant height, soybean varieties with low stem elongation plasticity might perform better in intercropping systems. A previous study of climbing plants grown under deep shade found that those plants with a higher capacity to intercept light were more abundant (Valladares et al., 2011), consistent with our finding that light capture contributed more to growth when soybeans could not escape from shade. In wheat/maize and wheat/cotton intercropping, although the leaf area of intercropped wheat and maize was lower compared with that of wheat and maize grown as single crops, the productivity of the intercrops was still increased by increased light interception (Zhang et al., 2008; Gou et al., 2017). In short, when selecting soybean varieties for intercropping, the focus should be on those traits related to light capture, such as leaf area per plant and leaflet area.

## Conclusion

A suite of shade responses for soybean varieties was documented. The biomass of soybean seedlings was positively correlated with leaf area per plant and stem diameter under both shade and full-light conditions. Although plant height increased significantly under shade, it was unrelated to the changes of biomass in this study. The top three most important explanatory variables of plasticity of biomass were leaf area, leaf area ratio and leaflet area. Plasticity of biomass was positively correlated with plasticity of leaf area and leaflet area and negatively correlated with plasticity of plant height, but it was not associated with plasticity of photosynthetic rate. These results suggest that increasing the capacity to capture light by increasing leaf area, rather than increasing the capacity to search for light by elongating the stem or by increasing light conversion in the leaf mesophyll, was more vital for light utilization by soybean seedlings grown under shade. Increasing light capture via the production of larger leaves gave rise to higher biomass accumulation in seedlings under shade. Therefore, selection and breeding of soybean varieties for future intercropping systems should focus on traits contributing to light capture, such as the production of more and larger leaves.

## Data availability statement

The original contributions presented in this study are included in the article/Supplementary material, further inquiries can be directed to the corresponding author.

## Author contributions

YW, WG, and WY designed the experiments. YW performed the experiments and data collection and wrote the manuscript. All authors contributed to the article and approved the submitted version.
