# Supplementary material for: Morphological and physiological variation of soybean seedlings in response to shade
Source: Front Plant Sci. 2022 Oct 6;13:1015414. doi: 10.3389/fpls.2022.1015414 (PMC9583947; doi:10.3389/fpls.2022.1015414)
Supplement: Supplementary file 1 [file Data_Sheet_1.docx]

**Table S1** The names of the soybean varieties used in this study.

| Experiment | ID | Name | Experiment | ID | Name |
| --- | --- | --- | --- | --- | --- |
| 1 | 1 | Qingdou 1 | 2 | 1 | **Qianshanwudou** |
|  | 2 | **Gongxuan 1** |  | 2 | Nan256-1 |
|  | 3 | Jiandebaimaodou |  | 3 | Xiaohuangdou |
|  | 4 | Jianyangjiuyuehuang 1 |  | 4 | Caidou |
|  | 5 | Jianyanglvpidou |  | 5 | Dahuangzhu |
|  | 6 | **Texuan 13** |  | 6 | **Guixia 3** |
|  | 7 | Dazhou 1 |  | 7 | Guang 15 |
|  | 8 | **Qianshanwudou** |  | 8 | Nandou021-1 |
|  | 9 | Nan032-4 |  | 9 | Dayuandou |
|  | 10 | Pingwubean 2 |  | 10 | **Texuan 13** |
|  | 11 | Chongmingbaimaobayueyi |  | 11 | Gongqiudou04-2 |
|  | 12 | **Nandou12** |  | 12 | **Nandou 12** |
|  | 13 | Gongdou 2 |  | 13 | **Gongxuan 1** |
|  | 14 | Jianyangjiuyuehuang 2 |  | 14 | Bayuehuang |
|  | 15 | Niufobean |  |  |  |
|  | 16 | Xiaobaimao |  |  |  |
|  | 17 | Zihuadou |  |  |  |
|  | 18 | Yongshengheidou | |  |  |
|  | 19 | Yaanheidou | |  |  |
|  | 20 | **Guixia 3** |  |  |  |

The soybean varieties used in both experiments were tagged in red bold.

**Table S2** Correlation coefficients for traits averaged across 20 soybean varieties grown under shade and full-light conditions.

|  | | CK→ |  |  |  |  |  |  |  |  |  |  |  |  |  |  | |  | |  | | |  | |  | |  | |  |  |
| --- | --- | --- | --- | --- | --- | --- | --- | --- | --- | --- | --- | --- | --- | --- | --- | --- | --- | --- | --- | --- | --- | --- | --- | --- | --- | --- | --- | --- | --- | --- |
|  | |  | BMS | f_S_ | fp | f_L_ | LA | LAR | PHT | L_1_ | L_2_ | L_3_ | DMT | LLA | LLN | LMA | | P_N500_ | | P_N1200_ | | | Chl | | Chl a/b | | C | | N |  |
| SH↓ | | BMS | — | 0.262 | 0.056 | -0.349 | 0.859** | -0.190 | 0.394 | 0.496* | 0.291 | 0.572** | 0.701** | 0.783** | 0.284 | -0.002 | | -0.132 | | 0.170 | | | 0.055 | | -0.063 | | -0.001 | | 0.083 |  |
|  | | f_S_ | 0.268 | — | -0.586** | -0.899** | 0.090 | -0.231 | 0.721** | 0.355 | 0.085 | 0.439 | 0.112 | -0.238 | 0.399 | -0.274 | | -0.139 | | 0.245 | | | 0.331 | | -0.213 | | -0.277 | | -0.202 |  |
|  | | fp | 0.224 | -0.247 | — | 0.173 | 0.220 | 0.118 | -0.205 | -0.428 | -0.122 | 0.007 | 0.261 | 0.360 | -0.047 | -0.047 | | -0.102 | | -0.308 | | | 0.005 | | 0.136 | | -0.035 | | -0.029 |  |
|  | | f_L_ | -0.362 | -0.925** | -0.140 | — | -0.178 | 0.190 | -0.657** | -0.195 | -0.039 | -0.451* | -0.217 | 0.102 | -0.389 | 0.358 | | 0.224 | | -0.131 | | | -0.406 | | 0.185 | | 0.356 | | 0.261 |  |
|  | | LA | 0.860** | 0.216 | -0.003 | -0.260 | — | 0.333 | 0.266 | -0.362 | -0.165 | 0.313 | 0.696** | 0.645** | 0.575** | -0.200 | | -0.008 | | 0.278 | | | 0.201 | | -0.050 | | -0.187 | | 0.036 |  |
|  | | LAR | 0.209 | -0.064 | -0.095 | 0.129 | 0.675** | — | 0.018 | -0.336 | -0.248 | 0.106 | 0.345 | 0.290 | 0.562** | -0.393 | | 0.149 | | 0.139 | | | 0.263 | | 0.086 | | -0.367 | | -0.130 |  |
|  | | PHT | 0.357 | 0.732** | -0.404 | -0.672** | 0.298 | -0.157 | — | 0.557* | 0.690** | 0.769** | -0.204 | 0.064 | 0.482* | -0.206 | | -0.073 | | 0.368 | | | 0.310 | | -0.071 | | -0.211 | | -0.070 |  |
|  | | L_1_ | -0.262 | 0.540* | -0.290 | -0.500* | 0.418 | -0.066 | 0.196 | — | 0.481* | 0.665** | 0.047 | 0.217 | 0.416 | 0.085 | | -0.316 | | 0.047 | | | 0.061 | | -0.038 | | 0.119 | | 0.098 |  |
|  | | L_2_ | -0.049 | 0.476* | -0.512* | -0.303 | 0.220 | -0.131 | -0.038 | 0.628** | — | 0.776** | -0.194 | 0.111 | 0.253 | -0.071 | | -0.157 | | 0.135 | | | 0.265 | | 0.006 | | -0.090 | | 00.01 |  |
|  | | L_3_ | 0.353 | 0.624** | -0.336 | -0.577** | 0.546* | -0.022 | 0.418 | 0.270 | 0.301 | — | 0.147 | 0.235 | 0.529* | -0.173 | | -0.179 | | 0.273 | | | 0.449* | | -0.064 | | -0.175 | | 0.002 |  |
|  | | DMT | 0.688** | -0.087 | 0.353 | -0.085 | 0.724** | 0.070 | 0.009 | -0.570** | -0.347 | 0.088 | — | 0.656** | 0.098 | 0.112 | | 0.042 | | 0.086 | | | 0.085 | | -0.115 | | 0.116 | | 0.260 |  |
|  | | LLA | 0.633** | -0.003 | 0.304 | -0.161 | 0.614** | -0.254 | -0.362 | -0.300 | 0.050 | 0.176 | 0.609** | — | -0.179 | 0.053 | | -0.224 | | -0.048 | | | -0.269 | | 0.056 | | 0.036 | | 0.047 |  |
|  | | LLN | 0.320 | 0.283 | -0.244 | -0.212 | 0.467* | 0.480* | 0.758** | -0.202 | -0.352 | 0.220 | 0.229 | -0.262 | — | -0.348 | | 0.277 | | 0.572** | | | 0.518* | | -0.107 | | -0.313 | | 0.048 |  |
|  | | LMA | -0.502* | -0.196 | -0.090 | 0.236 | -0.568** | -0.364 | -0.413 | 0.024 | -0.069 | -0.034 | -0.200 | -0.159 | -0.508* | — | | -0.011 | | -0.044 | | | -0.636** | | -0.092 | | 0.996** | | 0.771** |  |
|  | | P_N500_ | -0.087 | 0.172 | 0.209 | -0.258 | -0.061 | -0.004 | 0.040 | -0.098 | -0.387 | -0.305 | 0.024 | -0.240 | 0.146 | -0.023 | | — | | 0.787** | | | 0.202 | | -0.181 | | 0.015 | | 0.366 |  |
|  | | P_N1200_ | 0.229 | 0.225 | 0.434 | -0.400 | 0.300 | 0.242 | 0.077 | -0.249 | -0.254 | -0.212 | 0.414 | 0.054 | 0.201 | -0.194 | | 0.691** | | — | | | 0.210 | | -0.388 | | -0.026 | | 0.387 |  |
|  | | Chl | 0.345 | 0.260 | 0.242 | -0.360 | 0.488* | 0.418 | 0.272 | 0.141 | 0.201 | 0.152 | 0.204 | 0.111 | 0.351 | -0.743** | | 0.296 | | 0.574** | | | — | | -0.320 | | -0.620** | | -0.274 |  |
|  | | Chl a/b | -0.082 | -0.200 | -0.242 | 0.299 | 0.146 | 0.388 | -0.019 | -0.459* | -0.355 | -0.413 | 0.223 | -0.015 | 0.278 | 0.125 | | -0.024 | | -0.063 | | | 0.042 | | — | | -0.121 | | -0.282 |  |
|  | | C | -0.497* | -0.234 | -0.091 | 0.275 | -0.571** | -0.368 | -0.419 | 0.019 | -0.006 | -0.049 | -0.198 | -0.190 | -0.506* | 0.973** | | 0.055 | | -0.131 | | | -0.682** | | 0.102 | | — | | 0.795** |  |
|  | | N | -0.211 | -0.018 | -0.051 | 0.038 | -0.246 | -0.169 | -0.059 | -0.030 | 0.113 | 0.108 | -0.106 | 0.007 | -0.214 | 0.639** | | 0.052 | | 0.019 | | | -0.416 | | 0.199 | | 0.675** | | — |  |
|  | | Correlation coefficients under Ck are in listed right-upper panel, and correlation coefficients under SH are listed in left-lower panel. | | | | | | | | | | | | | |  | |  | |  | | |  | |  | |  | |  |  |
|  | | *, ** represent significant difference at 0.05 and 0.01 levels, respectively. | | | | | | | |  |  |  |  |  |  |  | |  | |  | | |  | |  | |  | |  |  |
| BMS, biomass (g); f_S_, fraction of dry mass in stem (%); f_P_, fraction of dry mass in petiole (%); f_L_, fraction of dry mass in lamina (%); LA, leaf area per plant (cm^2^); | | | | | | | | | | | | | | | | | | | |  |  | |  | |  | |  | | |  |
| LAR, leaf area ratio (m^2^ kg^-1^); PHT, plant height (cm); L_1_, first internode length (cm); L_2_, second internode length (cm); L_3_, third internode length (cm); | | | | | | | | | | | | | | | |  | |  | |  |  | |  | |  | |  | | |  |
| DMT, diameter (mm); LLA, leaflet area (cm^2^); LLN, leaflet number ; LMA, leaf mass per unit area (g m^-2^); P_N500_, net photosynthetic rate per unit area at 500μmol m^-2^ s^-1^ , (μmol m^-2^ s^-1^); | | | | | | | | | | | | | | | | | | | | |  | |  | |  | |  | | |  |
| P_N1200_, net photosynthetic rate per unit area at 1200μmol m^-2^ s^-1^ , (μmol m-2 s-1); Chl, chlorophyll content per unit dry mass (mg g^-1^); Chl a/b, chlorophyll a/b ratio; C, carbon content per unit area (g m^-2^);  N, nitrogen content per unit area (g m^-2^); | | | | | | | | | | | | | | | | | | | | | | | | | | | | | | |

| **Table S3** Correlation coefficients for plasticity in response to shade under shading net for all investigated traits averaged across 20 soybean varieties. | | | | | | | | | | | | | | | | | |  |  |  |  |
| --- | --- | --- | --- | --- | --- | --- | --- | --- | --- | --- | --- | --- | --- | --- | --- | --- | --- | --- | --- | --- | --- |
|  | BMS | f_S_ | fp | f_L_ | LA | LAR | PHT | L_1_ | L_2_ | L_3_ | DMT | LLA | LLN | LMA | P_N500_ | P_N1200_ | Chl | Chl a/b | C | N |  |
| BMS | — | 0.039 | -0.002 | -0.038 | 0.852** | 0.071 | -0.354 | -0.280 | 0.039 | -0.148 | 0.680** | 0.563** | 0.502* | 0.065 | -0.130 | 0.033 | 0.084 | 0.145 | 0.119 | 0.043 |  |
| f_S_ |  | — | 0.047 | -0.930** | -0.189 | 0.431 | 0.587** | 0.028 | 0.059 | 0.560* | 0.010 | 0.287 | -0.441 | -0.122 | -0.215 | -0.242 | 0.168 | 0.019 | -0.161 | -0.128 |  |
| fp |  |  | — | 0.322 | -0.099 | 0.250 | 0.128 | -0.071 | 0.373 | 0.325 | -0.035 | 0.023 | -0.057 | -0.273 | 0.138 | 0.450* | -0.119 | -0.434 | -0.267 | -0.377 |  |
| f_L_ |  |  |  | — | 0.143 | -0.317 | -0.510* | -0.052 | 0.081 | -0.412 | -0.022 | -0.264 | 0.396 | 0.015 | 0.254 | 0.395 | -0.203 | -0.177 | 0.055 | -0.017 |  |
| LA |  |  |  |  | — | -0.422 | -0.445* | -0.228 | -0.030 | -0.361 | 0.719** | 0.478* | 0.772** | -0.026 | -0.119 | 0.077 | 0.069 | 0.093 | 0.038 | 0.101 |  |
| LAR |  |  |  |  |  | — | 0.293 | -0.099 | 0.114 | 0.401 | -0.167 | -0.122 | -0.469* | 0.188 | 0.098 | -0.073 | 0.109 | 0.060 | 0.153 | -0.045 |  |
| PHT |  |  |  |  |  |  | — | -0.136 | -0.095 | 0.236 | -0.120 | -0.032 | -0.420 | -0.106 | -0.024 | -0.015 | -0.248 | -0.187 | -0.130 | -0.055 |  |
| L_1_ |  |  |  |  |  |  |  | — | 0.117 | 0.114 | -0.176 | -0.077 | -0.188 | -0.029 | 0.010 | 0.072 | 0.287 | -0.222 | -0.107 | 0.088 |  |
| L_2_ |  |  |  |  |  |  |  |  | — | 0.367 | 0.177 | -0.053 | -0.080 | 0.120 | 0.034 | 0.016 | 0.455* | -0.101 | 0.121 | -0.089 |  |
| L_3_ |  |  |  |  |  |  |  |  |  | — | -0.135 | 0.035 | -0.361 | 0.055 | -0.044 | 0.191 | 0.393 | -0.180 | 0.049 | -0.072 |  |
| DMT |  |  |  |  |  |  |  |  |  |  | — | 0.335 | 0.580** | 0.105 | -0.255 | -0.127 | 0.053 | -0.038 | 0.160 | 0.242 |  |
| LLA |  |  |  |  |  |  |  |  |  |  |  | — | 0.043 | -0.342 | -0.326 | -0.104 | -0.067 | 0.296 | -0.316 | -0.238 |  |
| LLN |  |  |  |  |  |  |  |  |  |  |  |  | — | 0.165 | 0.197 | 0.283 | 0.160 | -0.034 | 0.216 | 0.285 |  |
| LMA |  |  |  |  |  |  |  |  |  |  |  |  |  | — | 0.276 | 0.222 | 0.555* | -0.103 | 0.990** | 0.856** |  |
| P_N500_ |  |  |  |  |  |  |  |  |  |  |  |  |  |  | — | 0.658** | 0.155 | -0.147 | 0.225 | 0.075 |  |
| P_N1200_ | |  |  |  |  |  |  |  |  |  |  |  |  |  |  | — | 0.105 | -0.550* | 0.227 | 0.119 |  |
| Chl |  |  |  |  |  |  |  |  |  |  |  |  |  |  |  |  | — | 0.035 | 0.533* | 0.472* |  |
| Chl a/b | |  |  |  |  |  |  |  |  |  |  |  |  |  |  |  |  | — | -0.084 | -0.132 |  |
| C |  |  |  |  |  |  |  |  |  |  |  |  |  |  |  |  |  |  | — | 0.834** |  |
| N |  |  |  |  |  |  |  |  |  |  |  |  |  |  |  |  |  |  |  | — |  |
| *, ** represent significant difference at 0.05 and 0.01 levels, respectively. | | | | | | | | |  |  |  |  |  |  |  |  |  |  |  |  |  |


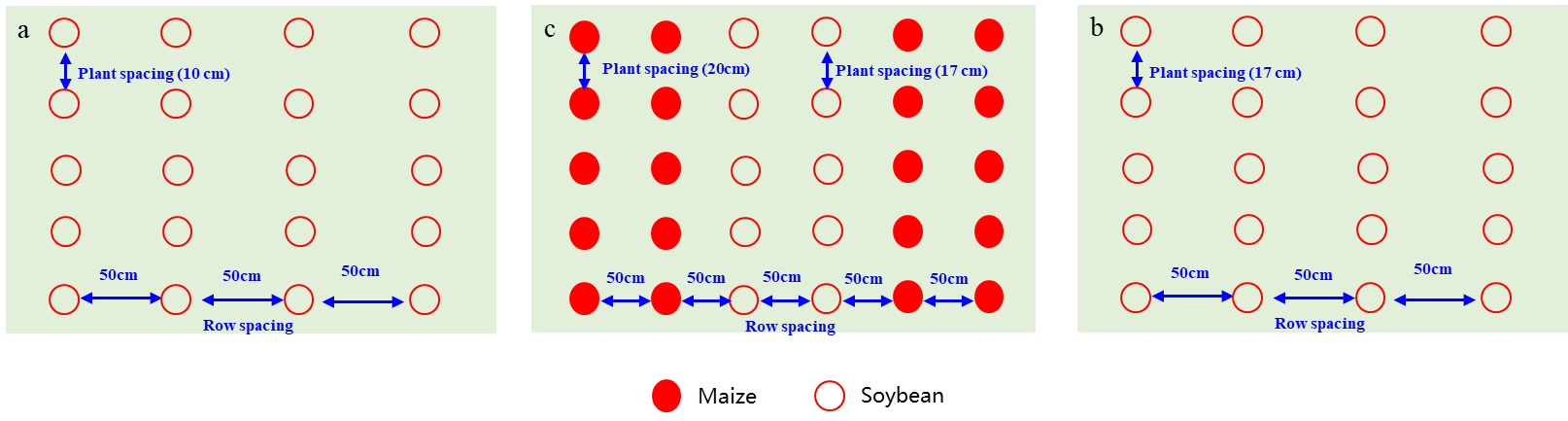


**Fig. S1** Schematics of the experiment design. a, full sunlight and shade in 2014; b, maize-soybean relay strip intercropping in 2015; c, soybean monoculture in 2015.

**Fig. S2** The weather condition (rainfall and temperature) of two years. a, 2014; b, 2015.


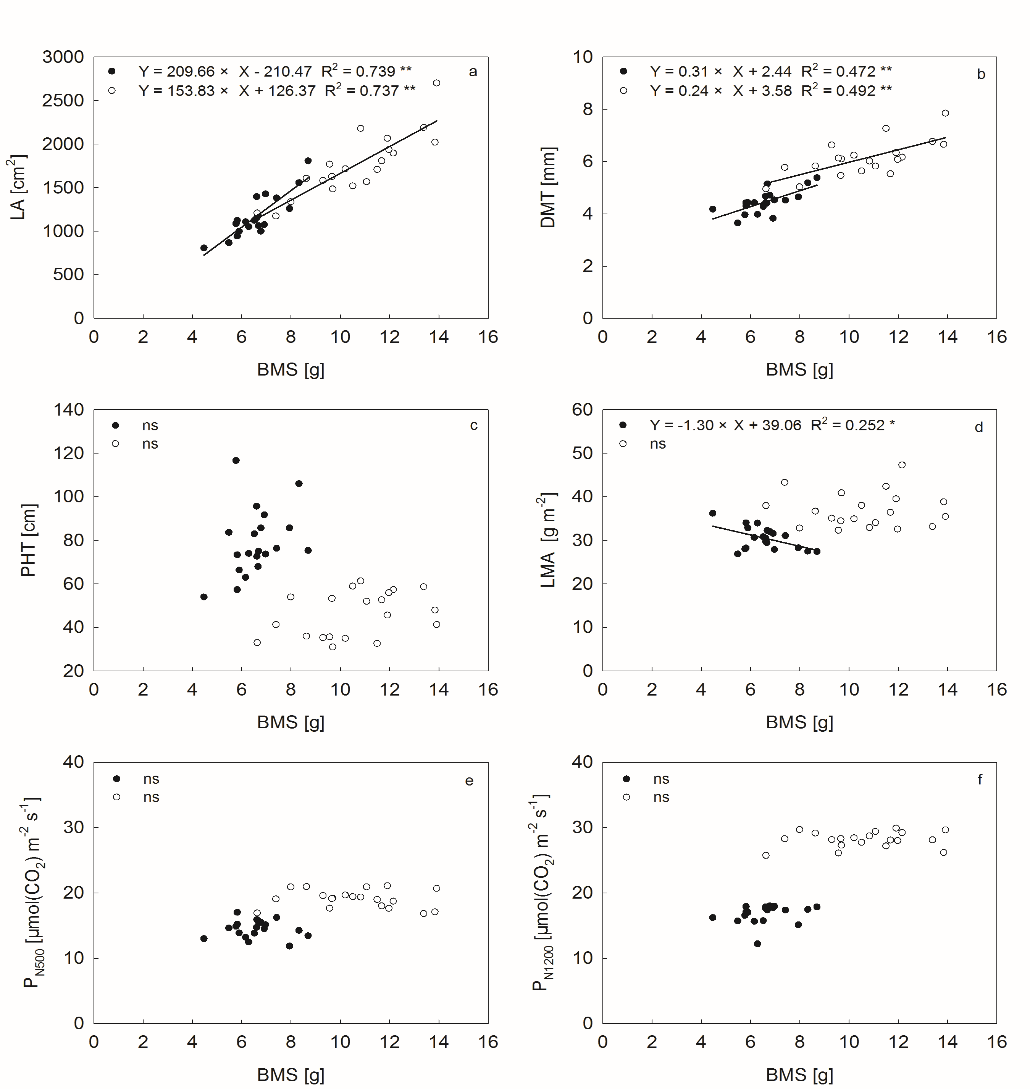


**Fig. S3** Relationship between biomass (BMS) and leaf area per plant (LA, a), stem diameter (DMT, b), plant height (PHT, c), leaf mass per unit area (LMA, d), and photosynthetic rate at 500 μmol m^-2^ s^-1^ (P_N500_, e) and 1200 μmol m^-2^ s^-1^ (P_N1200_, f) for 20 soybean varieties grown under shade (filled circles) and full-light (open circles) conditions in 2014. The regression equation and coefficient of determination (R^2^) are shown. *, **: significant difference at the 0.05 and 0.01 levels, respectively; ns: not significant.


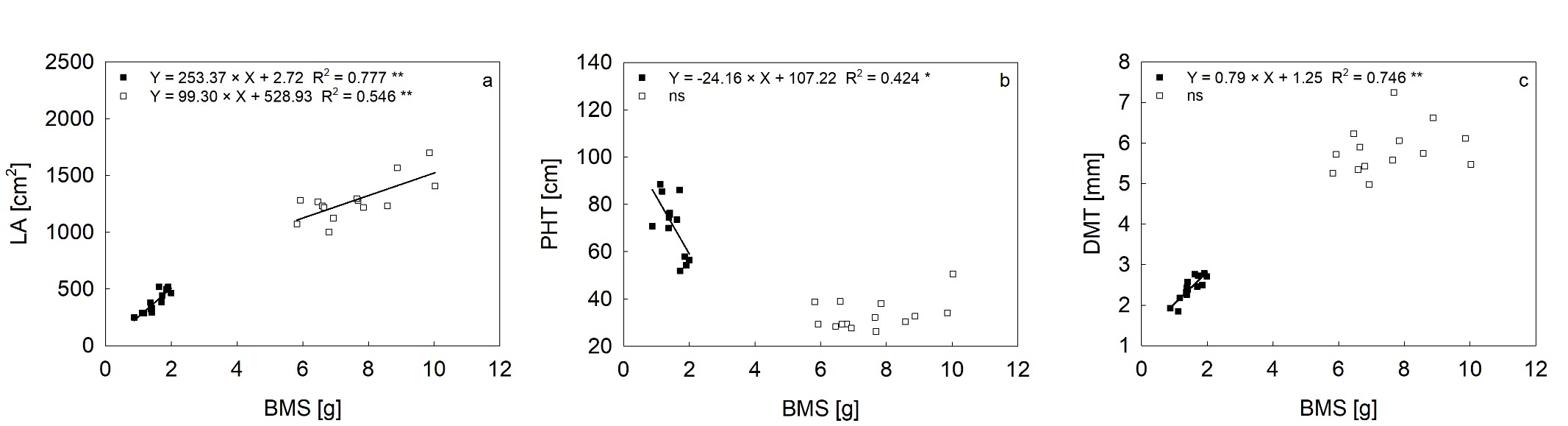


**Fig. S4** Relationship between biomass (BMS) and leaf area per plant (LA, a), plant height (PHT, b), and stem diameter (DMT, c) for 14 soybean varieties grown under shade in intercropping (filled squares) and full-light in sole cropping (open squares) conditions in 2015. The regression equation, coefficient of determination (R^2^) and the coefficients of correlation (r) are shown. *, **: significant difference at the 0.05 and 0.01 levels, respectively; ns; not significant.

**Table S4** Yield and yield components of 14 soybean varieties in 2014. Different letters in the same column between treatment within one variety are significantly different at the 0.05 probability level.

| Variety number | Treatment | Pod number  per plant | Seed number  per plant | 100 seed weight  (g) | yield per plant  (g) |
| --- | --- | --- | --- | --- | --- |
| 1 | Full light | 22.26±0.67a | 33.25±0.97a | 23.07±0.07a | 7.67±0.25a |
|  | Shade | 14.33±0.86b | 21.05±1.30b | 21.03±1.17b | 4.40±0.10b |
| 2 | Full light | 42.79±3.16a | 65.58±3.12a | 17.57±0.22a | 11.52±0.54a |
|  | Shade | 28.27±2.10b | 44.64±2.11b | 17.75±0.13a | 7.92±0.14b |
| 3 | Full light | 48.42±1.99a | 80.82±2.74a | 17.13±0.49a | 13.82±0.24a |
|  | Shade | 34.48±1.57b | 56.21±2.46b | 16.82±0.49b | 9.45±0.43b |
| 4 | Full light |  |  |  |  |
|  | Shade |  |  |  |  |
| 5 | Full light | 38.34±0.97a | 64.16±1.56a | 20.05±0.44a | 12.85±0.17a |
|  | Shade | 29.54±1.30b | 49.27±2.84b | 20.20±0.67a | 9.96±0.71b |
| 6 | Full light | 32.37±1.52a | 47.98±1.81a | 22.71±0.10a | 10.89±0.37a |
|  | Shade | 24.22±1.22b | 36.56±1.20b | 19.72±0.53b | 7.22±0.36b |
| 7 | Full light | 45.64±0.33a | 79.39±1.37a | 20.13±0.88a | 15.95±0.43a |
|  | Shade | 31.01±1.30b | 51.52±1.77b | 19.48±0.84a | 10.01±0.24b |
| 8 | Full light | 42.93±1.45a | 70.68±3.15a | 14.09±0.71a | 9.94±0.56a |
|  | Shade | 28.50±0.33b | 46.86±2.44b | 13.07±0.43b | 6.14±0.46b |
| 9 | Full light | 62.11±1.31a | 104.48±2.56a | 17.30±0.36a | 18.08±0.58a |
|  | Shade | 49.04±1.65b | 80.94±1.84b | 17.46±0.59a | 14.12±0.23b |
| 10 | Full light | 48.43±2.63a | 79.00±2.86a | 14.65±0.41a | 11.57±0.54a |
|  | Shade | 36.15±1.21b | 55.79±1.81b | 14.49±0.21a | 8.08±0.16b |
| 11 | Full light | 80.82±1.82a | 137.33±2.34a | 13.92±0.04a | 19.12±0.29a |
|  | Shade | 62.88±1.36b | 103.49±2.70b | 14.19±0.14a | 14.67±0.28b |
| 12 | Full light | 33.06±2.20a | 51.14±4.19a | 20.62±0.22a | 10.54±0.86a |
|  | Shade | 17.37±1.66b | 27.48±2.30b | 20.93±0.63a | 5.72±0.30b |
| 13 | Full light | 60.74±1.94a | 104.16±2.26a | 19.05±0.22a | 19.85±0.52a |
|  | Shade | 46.86±0.87b | 78.71±0.51b | 20.13±0.08a | 15.85±0.08b |
| 14 | Full light | 62.43±2.11a | 106.12±2.65a | 16.82±0.70a | 17.82±0.31a |
|  | Shade | 44.33±1.80b | 74.84±3.33b | 17.90±0.45a | 13.38±0.55b |
| 15 | Full light | 65.31±1.11a | 106.09±1.28a | 13.99±0.45a | 14.85±0.61a |
|  | Shade | 41.85±1.80b | 68.46±1.49b | 14.44±0.14a | 9.89±0.31b |
